# Endothelial Dysfunction, a Marker of Atherosclerosis, Is Independent of Metabolic Syndrome in NAFLD Patients

**DOI:** 10.1155/2020/1825142

**Published:** 2020-07-17

**Authors:** Jimmy Narayan, Haribhakti Seba Das, Preetam Nath, Ayaskanta Singh, Debakanta Mishra, Pradeep Kumar Padhi, Shivaram Prasad Singh

**Affiliations:** ^1^Department of Gastroenterology, IMS & SUM Hospital, SOA University, Bhubaneswar, Odisha 751003, India; ^2^Department of Gastroenterology, SCB Medical College & Hospital, Cuttack, Odisha 753001, India; ^3^Department of Gastroenterology, KIMS Hospital, KIIT University, Bhubaneswar, Odisha 751016, India

## Abstract

**Background:**

The study was designed to assess cardiovascular risk factors flow-mediated dilatation % (FMD%) and carotid intima-media thickness (CIMT) in NAFLD.

**Methods:**

126 NAFLD subjects and 31 chronic hepatitis B (CHB) controls were studied. Measuring carotid intima-media thickness (CIMT) and the flow-mediated dilatation % (FMD%) by brachial artery Doppler ultrasound were used to assess atherosclerosis. The risk of cardiac events at 10 years (ROCE 10) was estimated by the Prospective Cardiovascular Munster Study (PROCAM) score.

**Results:**

58 of 126 NAFLD have coexistent metabolic syndrome. Mean CIMT was 0.73 ± 0.041 mm among NAFLD with MS, 0.66 ± 0.016 mm among NAFLD without MS, and 0.66 ± 0.037 in controls CHB patients. FMD% in NAFLD with MS was 10.43 ± 3.134%, but was 8.56 ± 3.581% in NAFLD without MS and 17.78 ± 6.051% in controls. PROCAM score of NAFLD with MS was 46.95 ± 6.509 while in NAFLD without MS was 38.2 ± 3.738. Controls had a PROCAM score of 38.13 ± 5.755. ROCE 10 in NAFLD with MS was 13.64 ± 8.568 while NAFLD without MS was 5.55 ± 1.949. Controls have a ROCE 10 of 5.95 ± 3.973. Post hoc analysis showed CIMT was dependent upon MS while FMD% was different between all subgroups hence independent of metabolic syndrome.

**Conclusion:**

The markers of endothelial dysfunction are significantly higher in patients with NAFLD than controls.

## 1. Introduction

Nonalcoholic fatty liver disease (NAFLD) includes steatosis to steatohepatitis (NASH) [1]. NASH can progress on to cirrhosis and rarely to hepatocellular carcinoma (HCC) [2–4]. Moreover, NAFLD is one of the most common liver disorders in both developed and developing nations. Prevalence of NAFLD is estimated to be 15-35% in western countries [5] while it is 8-40% in Asian countries [6–9].

NAFLD, obesity, type 2 diabetes mellitus (T2DM), and dyslipidemia frequently coexist. NAFLD is now considered a part of the spectrum of metabolic syndrome (MS). Increased risk for cardiovascular disease is associated with NAFLD. Patients with MS were approximately 1.5–2 times more likely to develop coronary artery disease (CAD) than the controls as shown in the 3^rd^ National Health and Nutrition Examination Survey, and Atherosclerosis Risk in Communities (ARIC) study [10]. Carotid intima-media thickness (CIMT) and endothelial dysfunction studied by flow-mediated vasodilatation (FMD) are noninvasive methods to assess cardiovascular risk factors and atherosclerosis [11]. In India, limited literature is available to show a significant association between these two. Western data have demonstrated the association between increased CIMT and NAFLD. Some had predicted the risk of atherosclerosis and cardiovascular disease to be independent of MS [12–14].

The Prospective Cardiovascular Munster Study (PROCAM) score [15], Adult Treatment Panel III (ATP III) [16], or Framingham score can predict the risk of cardiovascular disease.

The aim of the study was to evaluate the prevalence of atherosclerosis by measuring the CIMT and flow-mediated vasodilation (FMD) in Indian patients with incidentally detected NAFLD and predicting the risk of cardiovascular disease by using the PROCAM score in NAFLD patients and its association with metabolic syndrome (MS).

## 2. Material and Methods

Single-center, case-control study was conducted in the Department of Gastroenterology, S.C.B. Medical College and Hospital, Cuttack, between January 2014 and December 2015.

NAFLD patients attending Gastroenterology OPD, SCB Medical College, Cuttack, were taken as cases. The diagnosis of NAFLD was made on the basis of ultrasonography. Cases fulfilling fatty liver definition criteria which were defined according to the American Gastroenterology Association are as follows: an increase in hepatic echogenicity taking renal echogenicity as a reference, the presence of enhancement, and lack of differentiation in periportal intensity and the vesicular wall due to great hyperechogenicity of the parenchyma.

Controls were taken as patients of chronic hepatitis B with persistent/intermittent elevation in the levels of serum transaminase level (ALT/AST) greater than the upper limit of normal (ULN) for at least 6 months with >6 months of HBsAg positivity.

Exclusion criteria were patient with alcohol intake of ≥20 g/d positive antibodies to hepatitis C virus (anti-HCV), positive autoimmune markers, abnormal iron profile drug usages such as corticosteroids, methotrexate or high-dose estrogens, and clinical or imaging features of cirrhosis of the liver.

All the subjects were explained thoroughly about the study. Those who signed informed consent were included in the study. Systemic examination was done. Body mass index (BMI), weight, waist circumference (WC), and hip circumference (HC) were measured in all patients. Complete blood count and routine biochemical investigations were performed in all subjects. The serum insulin level was assessed using the electrochemiluminescence method. IR was derived from FBG and plasma insulin (fasting insulin (*μ*U/mL) × FBG (mg/dL))/405) and measured as HOMA-IR value [17]. For assessing risk cardiovascular factors, electrocardiography (ECG) was done. Carotid intima-media thickness (CIMT) was assessed using a high-resolution B mode ultrasonography system (Phillips HD 11XE) with 7-12 MegaHertz transducer of both the right and left common carotid arteries at 3 points—base, midjunction, and just before bifurcation. The IMT is measured as the distance from the leading edge of the first echogenic line to the second echogenic line. The first echogenic line represents a luminal intimal interface while the second line represents the upper layer of intimal adventitia. IMTs were determined at the side of the greatest thickness and at two points, 1 cm upstream and 1 cm downstream. Six IMT measurements were done, and the mean was noted. For measuring brachial artery flow-mediated dilatation (FMD), rest and after reactive hyperemia diameter of the right brachial artery was measured. Tourniquet placed around the forearm to a pressure of 250 mmHg for 4 minutes and 30 seconds followed by a release increase flow was studied. The arterial diameter was measured at a fixed distance from an anatomical marker at rest and at 40, 60, and 80 seconds after the cuff release during systole. FMD% is calculated as FMD% = BADAV − BADB/BADB, where BADB is the brachial artery diameter at rest and postischemia is the BADAV. The risk of cardiac events at 10 years (ROCE 10) was estimated by the Prospective Cardiovascular Munster Study (PROCAM) score. The PROCAM score includes age, blood pressure, diabetes, cigarette smoking, total and low-density cholesterol, TGs, and family history of myocardial infarction.

### 2.1. Definitions

National Cholesterol Education Program-Adult Treatment Panel III (NCEP-ATP III) guidelines [18, 19] were used to define hypercholesterolemia. American Diabetes Association (ADA) criteria were used to define diabetes mellitus [20]. Normal ALT and AST values were taken as 0-40 IU/L. Hypertension was defined as blood pressure ≥140/90 mmHg or treatment with antihypertensive drugs. Having a normal fasting insulin level of 6-27 *μ*U/mL and a homeostasis model assessment of insulin resistance (HOMA-IR) value of ≥2.00 was taken as insulin resistance [21].

Obesity guidelines based on western populations is inaccurate for Asian individual; hence, the Asian Indians cut off was used in our study [22]. Overweight and obesity cut off was BMI ≥ 23 kg/m^2^ and ≥25 kg/m^2^, respectively. Metabolic syndrome diagnostic criteria were defined according to 2001 modified NCEP-ATP III-2 guidelines, which include any three of the following:

1. Abdominal obesity: men ≥90 cm, women ≥80 cm
2. Serum TG ≥150 mg/dL
3. HDL-C <40 mg/dL (male), <50 mg/dL (female)
4. Fasting blood glucose level ≥100 mg/dL
5. Blood pressure ≥130/≥85 mmHg

Statistical analysis was performed using SPSS 15.0. Student's *t*-test and Mann-Whitney *U* test were used for parametric and nonparametric continuous data, respectively, whereas chi-square test was applied for the categorical data. The strength of the correlation between quantitative variables was calculated by Pearson correlation coefficient, and Spearman correlation analysis was used for that of categorical variables. The final outcome variables were studied (NAFLD patients with MS, without MS and controls) using the Kruskal-Wallis test with subsequent pair-wise post hoc analysis. “*p*” value of <0.05 was defined as statistically significant.

## 3. Results

A total of 126 NAFLD subjects as cases and 31 chronic hepatitis B (CHB) patients as controls were enrolled in this study. NAFLD with metabolic syndrome were 58 subjects while the rest were NAFLD without metabolic syndrome. Among the controls (CHB), 19 (60%) were HBeAg negative, and the remaining 12 (40%) were HBeAg positive with a mean HBV DNA of 14.7 × 10^4^; standard deviation = 21.7 × 10 [3].

The male-female ratio was 2 : 1 among controls and 2.5 : 1 among cases; the gender difference between the two groups was comparable (*p* = 0.66). Baseline anthropometrical and biochemical data comparison between NAFLD and controls (CHB) is given in Table 1.

Baseline comparison between NAFLD with and without metabolic syndrome is depicted in Table 2, which showed that NAFLD with metabolic syndrome has higher BMI, waist, waist/hip, waist/height ratio, and higher systolic blood pressure as compared to NAFLD without metabolic syndrome. Furthermore, NAFLD subjects with metabolic syndrome had significantly higher fasting blood sugar, higher HOMA IR values, higher serum triglycerides, and lower HDL cholesterol than those without, whereas total, LDL, and VLDL cholesterol was comparable between these two groups. Besides, the LFT profile was comparable in both groups except for higher alkaline phosphatase and gamma-glutamyl peptidase in subjects with metabolic syndrome.

Mean carotid intima media thickness (right) was 0.73 + 0.046 mm among NAFLD with MS, 0.66 + 0.022 mm among NAFLD without MS, and 0.64 + 0.043 among controls chronic hepatitis B patients. Mean CIMT (left) was 0.74 + 0.04 mm among NAFLD with MS, 0.67 + 0.012 mm among NAFLD without MS, and 0.67 + 0.044 among controls chronic hepatitis B patients. Mean CIMT (mean) is 0.73 + 0.041 mm among NAFLD with MS, 0.66 + 0.016 mm among NAFLD without MS, and 0.66 + 0.037 among controls chronic hepatitis B patients. ANOVA showed significant difference between the groups and within the group for carotid intima media thickness (CIMT) (Table 3). Brachial artery dimension (BADB) at baseline is 3.92 + 0.228 mm among NAFLD with MS, 3.87 + 0.205 mm in NAFLD without MS, and 3.73 + 0.158 mm among chronic hepatitis B controls.

The markers of endothelial dysfunction such as carotid intima-media thickness (CIMT), brachial artery dimension at baseline (BADB), brachial artery dimension average after vasodilation (BADAV), flow-mediated vasodilation (FMD), PROCAM, and ROCE 10 score were compared in Table 3 and revealed a significant difference among the NAFLD with and without metabolic syndrome and the controls (*p* < 0.001, calculated by ANOVA). The scores were worse in patients with NAFLD and metabolic syndrome than those without and controls.

Post hoc analysis showed that carotid intima-media thickness is dependent upon metabolic syndrome while flow-mediated vasodilation is different between all subgroups, hence independent of metabolic syndrome (Table 4). A strong inverse correlation was found between the PROCAM score and FMD% (*p* < 0.0001); also, a positive correlation was found between the PROCAM score and CIMT. ROCE10 was found to have a strong positive correlation with the PROCAM score (*p* < 0.0001) and CIMT (Table 5).

## 4. Discussion

The association between NAFLD and metabolic syndrome is previously studied sparsely [23, 24]. Some considered it as a part of metabolic syndrome [25, 26]. In the present study, we studied atherosclerosis as measured by CIMT between patients of NAFLD and chronic hepatitis B controls. Targher et al. found similar results in NAFLD with CIMT (1.14 ± 0.20 vs. 0.82 ± 0.12 mm; *p* < 0.001) values higher than controls [12], and MS components were more frequent in NAFLD but the difference in CIMT observed between the groups was weakened following adjustment for individual metabolic syndrome components. Volzke et al. [27] postulated higher CIMT and more carotid plaques in fatty liver patients than controls (plaque prevalence rate 76.8% vs. 66.6%; *p* < 0.001). Brea et al. also reported higher CIMT values in NAFLD subjects which were independent of MS even after adjustment and logistic regression [28].

Duseja et al. found similar CIMT values (0.70 + 0.11) among NAFLD patients as compared to our study [3]. Also, in our study, no significant CIMT difference between NAFLD without metabolic syndrome and controls was found replicating the previous study of metabolic syndrome being the prime contributor to atherosclerosis.

In this study, NAFLD was associated with increased carotid IMT independently of other risk factors. Other factors of atherosclerosis may be playing an important role. Oxidative stress may play an important factor in the progression of NAFLD [29].

Brachial artery dimension at baseline and average dimension after vasodilation were higher among NAFLD as compared to chronic hepatitis B controls and, ANOVA analysis showed it to be a significant difference between NAFLD with and without metabolic syndrome. In our study, FMD% taken as a predictor of early atherosclerosis and endothelial dysfunction was found to be significantly lower in the NAFLD group (9.45 ± 3.49%) than in controls ((17.78 ± 3.49%) *p* < 0.0001). Villanova et al. also found similar results [13]. The FMD% in their study was remarkably higher in fatty liver (9.93%) as compared to NASH cases ((4.94%) *p* = 0.01). But after adjustment of confounding factors, they suggested the role of MS rather than NAFLD as the cause. Duseja et al. found FMD% among NAFLD group (9.7 ± 3.81%) different in comparison to controls ((17.03 ± 3.39%) *p* < 0.0001), but no difference in FMD% among patients of NAFLD with and without MS was found [3].

Thakur et al. [30] studied 40 nondiabetic subjects with NAFLD and 40 controls without NAFLD and measured atherosclerosis parameters and found similar results to our studies. NAFLD was found to be an independent predictor of CIMT and impaired FMD even after adjusting for different confounding factors.

Our study has interesting results while endothelial dysfunction, a marker of atherosclerosis and vascular instability, is independent of metabolic syndrome among fatty liver subjects supporting Thakur et al.'s study but carotid intima-media thickness (CIMT), an advance marker of cardiovascular risk factors, is dependent upon metabolic syndrome supporting Duseja's group. It could be due to a smaller sample size in Duseja et al.'s study and also different pathophysiology of endothelial dysfunction and CIMT thickness. More insight into this difference needs study based on molecular pathogenesis changes like studies implicating NO.

Increased atherosclerosis increases the risk of future cardiovascular events and also correlate with the severity of coronary atherosclerosis.

In our study, we observed that cardiovascular risk factor predicting scores (PROCAM, ROCE 10) were higher among fatty liver subjects which were dependent on the presence of MS. Villanova et al. found that ROCE10 moderately increased (*p* = 0.045) in patients with fatty liver [13]. Duseja et al. showed similar results among north Indian cohorts which show PROCAM and ROCE 10 dependent upon metabolic syndrome [3]. PROCAM score was found higher in fatty liver (27.50 ± 13.32) as compared to controls (20.10 ± 7.75) (*p* < 0.05). The PROCAM score of NAFLD and controls in our cohort is higher than the previous study which could be ascribed to the higher metabolic and cardiovascular risk factors in our group. Kessler et al. found a higher prevalence of myocardial infarction (66% and 50% for women and men, respectively) in the fatty liver while comparing to the normal population [31]. A prospective study involving 1221 participants by Hamaguchi et al. found an increased incidence of cardiovascular disease (coronary heart disease, ischaemic stroke, and cerebral hemorrhage) in 231 patients with the fatty liver as compared to 990 normal population without NAFLD [32]. We found a strong (*p* < 0.001) inverse correlation between the PROCAM score and FMD%. PROCAM score and CIMT show a positive correlation in fatty liver subjects. ROCE10 also shows a positive correlation (*p* < 0.0001) with the PROCAM score and CIMT. Duseja et al. have found similar results but they found a strong correlation between FMD and ROCE 10 which was absent in our study; probably larger sample size with more homogenous population would shed better light in this subject [3].

The practical implication of our study is that a reproducible assessment of cardiovascular risk factors which is the prime cause of mortality among NAFLD is studied.

Histopathology diagnosis for fatty liver diagnosis is a limitation of our study. In a recent study, NAFLD fibrosis score (NFS), as a marker of NAFLD, could identify patients at higher risk of CVD; this is a relevant finding [33]. Ultrasonography cannot identify fatty infiltration of the liver below 30% which makes liver biopsy as the gold standard to diagnose fatty liver but is not practical in this group. In our study, we have not assessed calorie intake, physical activity, sedentary lifestyle, and smoking habits which also have an impact on the cardiovascular risk factor.

## Figures and Tables

**Table 1:** Baseline anthropometrical and biochemical comparison between NAFLD and controls (chronic hepatitis B (CHB)).

Parameters	NAFLD (*n* = 126)	CHB (*n* = 31)	*p* value
Age	45.78 ± 11.67	46.55 ± 11.89	0.74
BMI	28.97 ± 6.33	27.02 ± 3.63	0.10
Waist circumference	100.83 ± 12.81	94.68 ± 8.32	0.01
Waist/hip ratio	0.99 ± 0.08	0.97 ± 0.08	0.27
Waist/height ratio	0.62 ± 0.1	0.61 ± 0.06	0.23
SBP	128.65 ± 21.05	126.9 ± 9.09	0.65
DBP	81.5 ± 11.6	82.1 ± 4.7	0.77
Total bilirubin	1.15 ± 3.03	0.79 ± 0.25	0.51
AST	34.02 ± 18.32	44.23 ± 20.29	0.08
ALT	47.71 ± 32.89	54.7 ± 31.93	0.07
Alkaline phosphatase	180.48 ± 71.24	220.42 ± 64.75	0.005
Total protein	7.9 ± 2.46	7.36 ± 0.56	0.45
Albumin	4.21 ± 0.28	4.19 ± 0.29	0.73
Globulin	3.75 ± 0.66	3.16 ± 0.55	0.42
GGT	34.56 ± 22.98	27.92 ± 26.58	0.17
Insulin	11.18 ± 7.13	8.14 ± 3.75	0.02
HOMA IR	3.17 ± 4.29	2.07 ± 1.39	0.01
FBS	112.8 ± 44.7	104.6 ± 17.14	0.32
2hour PPBS	171.8 ± 83.82	141.2 ± 42.11	0.05
Triglycerides	176.8 ± 86.73	183.4 ± 76.41	0.70
Total cholesterol (TC)	197.4 ± 47.19	213.3 ± 36.3	0.08
HDL	47.68 ± 18.43	44.65 ± 7.83	0.37
LDL	118.8 ± 33.1	131.9 ± 28.27	0.04
VLDL	35.9 ± 19.05	38.9 ± 15.68	0.41
LDL/HDL	2.82 ± 0.89	2.82 ± 0.48	0.99
TC/HDL	4.44 ± 1.06	4.77 ± 0.69	0.29

**Table 2:** Baseline clinical, anthropometrical, and biochemical comparison between NAFLD with MS and NAFLD without MS.

Parameters	NAFLD with MS (*n* = 58)	NAFLD without MS (*n* = 68)	*p* value
Age	47.55 ± 11.46	44.26 ± 11.71	0.11
BMI	33.78 ± 4.86	24.87 ± 4.21	<0.001
Waist circumference	113.24 ± 4.69	90.24 ± 6.36	<0.001
Waist/hip ratio	1.02 ± 0.08	0.96 ± 0.06	<0.001
Waist/height ratio	0.7 ± 0.06	0.56 ± 0.08	<0.001
SBP	134.5 ± 22.59	118.65 ± 13.26	<0.001
DBP	82.8 ± 12.73	79.4 ± 9.28	0.17
Total bilirubin	0.74 ± 0.23	1.51 ± 0.86	0.16
AST	32 ± 11.3	35.7 ± 22.7	0.25
Alkaline phosphatase	204.1 ± 69.7	160.5 ± 66.7	0.001
Total protein	7.19 ± 0.73	7.36 ± 0.56	0.125
Albumin	4.18 ± 0.23	4.23 ± 0.33	0.358
Globulin	3.03 ± 0.69	4.55 ± 0.55	0.42
GGT	39.23 ± 25.05	29.96 ± 19.91	0.03
Insulin	14.22 ± 7.93	8.03 ± 4.39	<0.001
HOMA IR	4.89 ± 5.7	1.71 ± 1.42	<0.001
FBS	126.59 ± 59.62	100.87 ± 19.29	0.002
2hour PPBS	197.4 ± 98.3	149.42 ± 61.06	0.001
Triglycerides	194.62 ± 103.4	161.68 ± 66.57	0.033
Total cholesterol (TC)	197.9 ± 45.59	197 ± 48.85	0.913
HDL	43.9 ± 9.03	51.68 ± 23.01	0.008
LDL	117.0 ± 36.93	120.49 ± 29.64	0.558
VLDL	39.21 ± 19.24	33.06 ± 18.56	0.073
LDL/HDL	2/88 ± 0.87	2.78 ± 0.87	0.629
TC/HDL	4.23 ± 1.1	4.68 ± 0.96	0.06

**Table 3:** Carotid intima-media thickness (CIMT), brachial artery dimension at baseline (BADB), brachial artery dimension average after vasodilation (BADAV), flow-mediated vasodilation (FMD), PROCAM, and ROCE 10 score in NAFLD (with and without MS) and CHB.

	NAFLD with MS (*n* = 58)	NAFLD without MS (*n* = 68)	CHB (*n* = 31)	*p* value^∗^
CMIT R	0.73 ± 0.05	0.66 ± 0.02	0.64 ± 0.04	<0.001
CIMT L	0.74 ± 0.04	0.67 ± 0.01	0.67 ± 0.04	<0.001
CIMT M	0.73 ± 0.04	0.66 ± 0.02	0.66 ± 0.04	<0.001
BADB	3.92 ± 0.23	3.87 ± 0.21	3.73 ± 0.16	<0.001
BADAV	4.32 ± 0.17	4.20 ± 0.18	4.39 ± 0.11	<0.001
FMD	10.43 ± 3.13	8.56 ± 3.58	17.78 ± 6.05	<0.001
PROCAM	46.95 ± 6.51	38.20 ± 3.74	38.13 ± 5.8	<0.001
ROCE 10	13.64 ± 8.57	5.95 ± 1.97	5.55 ± 1.94	<0.001

^∗^Calculated by ANOVA.

**Table 4:** Post hoc analysis of carotid intima-media thickness (CIMT) and flow-mediated vasodilation (FMD).

Variables	NAFLD with MS (*n* = 58)	NAFLD without MS (*n* = 68)	*p* value^∗^
CIMT	NAFLD without MS	NAFLD with MS	<0.001
	NAFLD without MS	CHB	0.976
	NAFLD with MS	CHB	<0.001
FMD	NAFLD without MS	NAFLD with MS	0.036
	NAFLD without MS	CHB	<0.001
	NAFLD with MS	CHB	<0.001

**Table 5:** Correlation between PROCAM score, CIMT, ROCE10, and FMD.

Variable 1	Variable 2	*p* value^∗^	Correlation
PROCAM	CIMT M	<0.001	Positive
PROCAM	FMD	<0.001	Inverse
PROCAM	ROCE 10	<0.001	Positive
CIMT M	ROCE 10	<0.001	Positive
ROCE 10	FMD	0.168	

## Data Availability

Data are available and will be provided on request.
